# A longitudinal study of serum insulin and glucose levels in relation to colorectal cancer risk among postmenopausal women

**DOI:** 10.1038/bjc.2011.512

**Published:** 2011-11-29

**Authors:** G C Kabat, M Y Kim, H D Strickler, J M Shikany, D Lane, J Luo, Y Ning, M J Gunter, T E Rohan

**Affiliations:** 1 Department of Epidemiology and Population Health, Albert Einstein College of Medicine, Bronx, NY 10461, USA; 2 Division of Preventive Medicine, Department of Medicine, University of Alabama at Birmingham, Birmingham, AL 35294, USA; 3 Department of Preventive Medicine, Stony Brook University Medical Center, Stony Brook, NY 11794, USA; 4 Department of Community Medicine, School of Medicine, West Virginia University, Morgantown, WV 26506, USA; 5 Department of Epidemiology and Community Health, School of Medicine, Virginia Commonwealth University Medical Center, Richmond, VA 23298, USA; 6 Department of Epidemiology and Biostatistics, School of Public Health, Imperial College London, Norfolk Place, London W2 1PG, UK

**Keywords:** serum insulin, glucose, homeostasis model assessment-insulin resistance, colorectal cancer

## Abstract

**Background:**

It is unclear whether circulating insulin or glucose levels are associated with increased risk of colorectal cancer. Few prospective studies have examined this question, and only one study had repeated measurements.

**Methods:**

We conducted a prospective study of colorectal cancer risk using the subsample of women in the Women's Health Initiative study whose fasting blood samples, collected at baseline and during follow-up, were analysed for insulin and glucose. Cox proportional hazards models were used to assess associations with colorectal cancer risk in both baseline and time-dependent covariates analyses.

**Results:**

Among 4902 non-diabetic women with baseline fasting serum insulin and glucose values, 81 incident cases of colorectal cancer were identified over 12 years of follow-up. Baseline glucose levels were positively associated with colorectal cancer and colon cancer risk: multivariable-adjusted hazard ratio (HR) comparing the highest (⩾99.5 mg dl^−1^) with the lowest tertile (<89.5 mg dl^−1^): 1.74, 95% confidence interval (CI) 0.97–3.15 and 2.25, 95% CI: 1.12–4.51, respectively. Serum insulin and homeostasis model assessment were not associated with risk. Analyses of repeated measurements supported the baseline results.

**Conclusion:**

These data suggest that elevated serum glucose levels may be a risk factor for colorectal cancer in postmenopausal women.

Obesity, diabetes, and physical inactivity show consistent positive associations with risk of colorectal cancer (Moghaddam *et al*, 2007; Huxley *et al*, 2009; Ning *et al*, 2010). They are also major determinants of insulin resistance, hyperinsulinaemia, hyperglycaemia, and the metabolic syndrome (McKeown-Eyssen, 1994; Giovannucci, 1995). Insulin has long been regarded as a possible mediator of the effects of obesity and physical inactivity on colorectal cancer risk (McKeown-Eyssen, 1994; Giovannucci, 1995; Strickler *et al*, 2001), since, in addition to its role in metabolism, insulin has mitogenic and anti-apoptotic activity (Koenuma *et al*, 1989; Bjork *et al*, 1993; Wu *et al*, 1995; Bruce and Corpet, 1996). Higher insulin levels may also increase the bioavailability of insulin-like growth factor-1 (IGF-1), a related mitogen that has been associated with increased risk of other cancers (Werner and LeRoith, 1996). Further, high glucose levels may increase colorectal cancer risk independently of insulin either by favouring the selection of malignant clones (Warburg, 1956) or by acting as a direct source of energy for neoplastic cells (McKeown-Eyssen, 1994).

Epidemiologic studies that have examined the association of colorectal cancer with circulating insulin and/or glucose levels have yielded conflicting results (Yamada *et al*, 1998; Schoen *et al*, 1999; Nilsen and Vatten, 2001; Trevisan *et al*, 2001; Palmqvist *et al*, 2003; Saydah *et al*, 2003; Jee *et al*, 2005; Tsushima *et al*, 2005; Limburg *et al*, 2006; Gunter *et al*, 2008; Stocks *et al*, 2011). Findings of studies that measured glycosylated haemoglobin (Saydah *et al*, 2003; Wei *et al*, 2005) or blood levels of C-peptide (Kaaks *et al*, 2000; Ma *et al*, 2004; Wei *et al*, 2005; Jenab *et al*, 2007; Otani *et al*, 2007), markers of glucose control and insulin production, respectively, have also been inconsistent. The inconsistent results may in part reflect differences in study design, age, and sex distributions of the populations studied, whether fasting blood samples were obtained, as well as the reliance in all but one study (Stocks *et al*, 2011) on a single, baseline, measurement of the study factors.

Therefore, we conducted a longitudinal study of colorectal cancer risk in which fasting serum insulin and glucose levels were measured at baseline and during follow-up in a subsample of participants in the Women's Health Initiative (WHI) clinical trials (CTs) and observational study (OS).

## Materials and methods

### Study population

The WHI is a large, prospective, multi-center study of factors affecting the health of postmenopausal women. It includes an OS (*N*=93 676) and CTs (*N*=68 132) of hormone therapy, dietary modification (low-fat dietary intervention), and calcium plus vitamin D supplementation (The Women's Health Initiative Study Group, 1998). Subjects were recruited between 1 October 1993 and 31 December 1998 at 40 clinical centres throughout the United States, largely through direct mailings. The eligibility requirements for the study included being postmenopausal, age between 50 and 79 years, a high likelihood of residing in the same residence for at least 3 additional years, and having provided written informed consent. The CTs had a number of additional eligibility requirements (Chlebowski *et al*, 2005). In general, eligible women were first invited to enroll in the CT component. Women who did not wish to be randomly assigned to an intervention or who were ineligible for the CT component were then invited to participate in the OS.

The present analysis is based on a 6% random sample of women in the CTs (*N*=4544) and a 1% sample of women in the OS (*N*=1062). All WHI participants had provided a fasting blood sample at baseline. Women in the 6% subsample additionally provided fasting blood samples at years 1, 3, and 6 of follow-up, and women in the 1% sample of women in the OS additionally provided fasting blood samples in year 3 (Howard *et al*, 2004). The 6% random sample was stratified by age, clinical centre, and hysterectomy status, with oversampling of minority groups to increase the numbers of Black, Hispanic, and Asian-Pacific women. All protocols and procedures were approved by Institutional Review Boards at participating institutions, and written informed consent was obtained from all participants. Of the 5606 women with blood samples, 5476 had a baseline glucose value and 5312 of these also had a baseline insulin value and were included in the present analysis (1059 from the OS and 4417 from the CT). We excluded from the analysis women who at baseline either had a diagnosis of diabetes, were taking diabetes medication, or who had a fasting blood glucose of ⩾126 mg dl^−1^ (8 cases and 556 non-cases), leaving 81 cases and 4821 non-cases for analysis.

Of the 5312 women with a baseline insulin measurement, the numbers with measurements during follow-up were 3480 (65.5%-year 1), 3049 (57.4%-year 3), and 2976 (56.0%-year 6). Of the 5476 women with a baseline glucose measurement, the numbers with measurements during follow-up were 3587 (65.5%-year 1), 3227 (58.9%-year 3), and 2980 (54.4%-year 6). The proportions were similar for different blood draws among cases and non-cases.

### Case ascertainment

Cancer outcomes were initially ascertained through either self-administered questionnaires at semi-annual visits (CTs) or through questionnaires that were mailed annually (OS). Potential cases were then confirmed by centralised review of pathology reports, discharge summaries, operative and radiology reports, and tumour registry abstracts by trained medical adjudicators. After the end of CT period (2005), outcomes in all women were identified on an annual basis by mailed questionnaires. The rate of confirmation of locally adjudicated outcomes by central adjudication was 94% for colorectal cancer (Curb *et al*, 2003).

### Laboratory methods

Fasting blood samples were collected with minimal stasis and maintained at 4 °C until plasma/serum was separated. Plasma/serum aliquots were then frozen at −70 °C and sent on dry ice to the central repository (Fisher BioServices, Rockville, MD, USA), where storage at −70 °C was maintained. In the CT, serum samples used for the insulin and glucose measurements went through one freeze/thaw cycle, while those from the OS went through at least two cycles. Since analyses were stratified on participation in the OS *vs* the CTs, samples of cases and non-cases within each cohort underwent similar processing. Serum glucose was measured using the hexokinase method on the Hitachi 747 (Boehringer Mannheim Diagnostics, Indianapolis, IN, USA) (Peterson and Young, 1968; Bergmeyer, 1974). Monthly inter-assay coefficients of variation (CV) were <2% for mean concentrations of 84 and 301 mg dl^−1^. Serum insulin was measured in a step-wise sandwich ELISA procedure on an ES 300 (BMD; Tietz, 1987). Monthly inter-assay CV were 4.7–9.5% and 3.2–7.9% at mean concentrations of 26.6 and 80.6 *μ*IU ml^−1^, respectively.

### Statistical analysis

Cox proportional hazards models were used to estimate hazard ratios (HRs) and 95% confidence intervals (95% CIs) for the associations of serum insulin and glucose with risk of colorectal cancer, with duration of follow-up (days) as the time scale. For these analyses, study participants were considered to be at risk from their date of enrollment until the date of diagnosis of their colorectal cancer, termination of follow-up (30 September 2010), loss to follow-up, withdrawal from the study, or death, whichever occurred first. Event times of participants who had not developed colorectal cancer by the end of follow-up, who had died, or who had withdrawn from the study before the end of follow-up, were censored.

We estimated the risk of colorectal cancer in association with baseline insulin and glucose levels, and with a measure of insulin resistance, the homeostasis model assessment-insulin resistance (HOMA-IR) index ((fasting insulin (*μ*IU ml^−1^) × fasting glucose (mg dl^−1^))/22.5) (Matthews *et al*, 1985). Tertiles of the three exposure variables were created based on their distribution in the 4902 subjects with insulin and glucose measurements and without diabetes. To increase the statistical power, we also used the continuous variables to estimate the HR per 1 mg dl^−1^ of insulin and glucose and per unit of HOMA-IR. Age-adjusted and multivariable-adjusted HRs and 95% CIs were computed. Covariates were selected for inclusion in the multivariate analysis if their addition to the model changed the parameter estimate of the study variables by >10%. The covariates included in the final model were age (years-continuous), body mass index (kg m^−2^-continuous), alcohol intake (drinks per week-continuous), physical activity (MET-hours per week-continuous), family history of colorectal cancer (no, yes), and ethnicity (white, black, other). In addition, dummy variables for participation in the OS or specific treatment arm of one of the CTs were included in all models. We carried out stratified analyses by stratifying BMI and physical activity at their median values (27.76 kg m^−2^ and 5.75 MET-hour per week, respectively) and treating glucose and insulin as continuous variables. Tests for trend were performed by assigning the median value to each category and modelling this variable as a continuous variable.

To minimise the possibility that observed associations were due the influence of the presence of preclinical disease on the exposure variables of interest, we carried out a sensitivity analysis by excluding cases diagnosed during the first 2 years following enrollment. In a second sensitivity analysis, we excluded women in the OS component of WHI, representing 19% of the total study population, because, compared with women in the CT, women in the OS had substantially lower body weight—a factor known to influence insulin and glucose levels.

In addition, we analysed the repeated measurements of insulin, glucose, and HOMA-IR as time-dependent covariates in Cox proportional hazards models (Gail, 1981). The associations of the average (mean) of the repeated measurements and measurements obtained 1–3 years, 2–4 years, and 3–5 years before the date of diagnosis of the case with risk of colorectal cancer were evaluated. Among cases, levels that were measured within 1 year of diagnosis were excluded from all analyses, since these values may have been influenced by the presence of subclinical disease. All *P*-values were two-sided.

## Results

During a median follow-up period of 11.9 years (0.05–15.4), a total of 81 colorectal cancer cases were ascertained in the cohort of 4902 non-diabetic women with fasting serum insulin and glucose values at baseline. Sixty-five cases had colon cancer; six had cancer of the rectosigmoid junction; and ten had rectal cancer. In all, 38 cases and 2372 non-cases were not in any of the CT intervention groups.

Compared with non-cases, on average cases were older, less physically active (as reflected by estimated metabolic equivalent tasks per week), and more likely to be non-Hispanic white (Table 1). In other respects, cases and non-cases were broadly similar.

Baseline serum glucose and insulin levels were modestly correlated (Pearson's correlation coefficient 0.31, *P*<0.0001) and baseline glucose showed a moderately strong association with HOMA-IR (correlation coefficient 0.49, *P*<0.0001), whereas insulin was strongly correlated with HOMA-IR (correlation coefficient 0.92, *P*<0.0001). Although levels of glucose and insulin at follow-up visits were correlated with baseline levels, the strength of these correlations decreased with increasing interval between measurements (data not shown).

In both age-adjusted and multivariable-adjusted analyses, baseline insulin levels and HOMA-IR were not associated with colorectal cancer risk (Table 2). However, in the age- and multivariable-adjusted analysis, baseline glucose was positively associated with risk: in the multivariable model, the HR for the highest (⩾99.5 mg dl^−1^) *vs* lowest tertile (<89.5 mg dl^−1^) was 1.74, 95% CI 0.97–3.15, *P* for trend 0.06. When the HRs for insulin and glucose were mutually adjusted, the HR for highest *vs* lowest tertile of insulin was 0.88, 95% CI: 0.47–1.65, *P* for trend 0.70, and that for glucose was 1.72, 95% CI: 0.94–3.15, *P* for trend 0.07. The HR per 1 mg dl^−1^ of glucose was 1.031, 95% CI: 1.009–1.054, *P* for trend=0.0066. When the analysis was restricted to cases of colon cancer (*N*=65), HRs for the middle and highest tertiles *vs* the lowest tertile of glucose were 1.72 (95% CI: 0.85–3.48) and 2.25 (95% CI: 1.12–4.51), respectively, *P* for trend 0.02, whereas insulin and HOMA-IR were not associated with risk. Further adjustment for total serum cholesterol did not affect the results (data not shown). The HR per 1 mg dl^−1^ of glucose was 1.034, 95% CI: 1.009–1.060, *P* for trend=0.0066.

The positive association of glucose with colorectal cancer was very similar in high and low BMI groups (⩾27.76 and <27.76 kg m^−2^): HR per mg dl^−1^ 1.029 (95% CI: 0.997–1.062, *P*=0.08) and 1.031 (95% CI: 1.001–1.063, *P*=0.04), respectively. Similar results were seen for colon cancer. HRs for glucose with colorectal cancer within strata of physical activity (dichotomised at the median of 5.75 MET-hour per week) were 1.039 (95% CI: 1.006–1.074, *P*=0.02) and 1.027 (95% 0.997–1.057, *P*=0.08), respectively. Similar results were seen for colon cancer. Neither insulin nor HOMA-IR was associated with risk.

In analyses excluding cases diagnosed within the first 2 years of follow-up (*N*=17), the associations were unchanged: HR of colorectal cancer for the highest *vs* lowest tertile of glucose was 1.81, 95% CI 0.93–3.51, *P* for trend 0.07. In the analysis restricted to women in the CT (10 OS cases excluded), insulin and HOMA-IR showed no association, but the association of glucose with colorectal cancer was strengthened: HRs for intermediate and highest tertiles relative to the lowest: 1.60, 95% CI: 0.82–3.14, and 2.10, 95% CI: 1.08–4.06, *P* for trend 0.03.

In the time-dependent covariates analysis, the average (mean) of the glucose measurements obtained during follow-up until the date of diagnosis of the case (excluding measurements within 1 year of diagnosis for cases) was associated with significantly increased risk of colorectal (76 cases) and colon cancer (61 cases): HR per 1 mg dl^−1^ of glucose 1.020, 95% CI 1.001–1.039, *P*=0.0384 for colorectal cancer and 1.021 (1.001–1.042, *P*=0.0423 for colon cancer, respectively. The analyses of glucose measured during various time intervals preceding diagnosis of colorectal cancer (1–3 years; 2–4 years; 3–5 years) also showed positive trends but were not statistically significant due to the smaller number of subjects with measurements obtained during these specific periods. As in the baseline analysis, no associations were seen with insulin or HOMA-IR.

## Discussion

The results of the present study suggest that baseline fasting serum glucose levels are positively associated with risk of colorectal cancer in postmenopausal women, whereas insulin and HOMA-IR are not associated with risk. The association was not affected by adjustment for insulin level or by the exclusion of cases diagnosed within the first 2 years following enrollment. Baseline glucose appeared to be more strongly associated with colon cancer than with all colorectal cancers. Analyses of the repeated measures obtained during follow-up using time-dependent covariates showed a statistically significant positive association of fasting glucose with colorectal and colon cancer, but power for the time-lagged models was diminished due to smaller available sample sizes.

Several previous studies have examined the association of circulating insulin and/or glucose levels with risk of colorectal cancer (Yamada *et al*, 1998; Schoen *et al*, 1999; Nilsen and Vatten, 2001; Trevisan *et al*, 2001; Palmqvist *et al*, 2003; Saydah *et al*, 2003; Jee *et al*, 2005; Tsushima *et al*, 2005; Limburg *et al*, 2006; Gunter *et al*, 2008; Stocks *et al*, 2011). Of five studies (Schoen *et al*, 1999; Palmqvist *et al*, 2003; Saydah *et al*, 2003; Limburg *et al*, 2006; Gunter *et al*, 2008) with results regarding insulin levels, only one (Schoen *et al*, 1999) showed evidence of a positive association after adjusting for covariates. Of nine studies that examined blood glucose levels in relation to colorectal cancer risk (Yamada *et al*, 1998; Schoen *et al*, 1999; Nilsen and Vatten, 2001; Trevisan *et al*, 2001; Tsushima *et al*, 2005; Limburg *et al*, 2006; Gunter *et al*, 2008; Stocks *et al*, 2011), one found a weak positive association (with *in-situ* carcinoma) (Yamada *et al*, 1998), two reported significant positive associations with colorectal cancer (Schoen *et al*, 1999; Trevisan *et al*, 2001), one reported a significant association in females but not in males (Nilsen and Vatten, 2001), whereas another reported a significant association in males but not in females (Jee *et al*, 2005), and four studies found no association (Tsushima *et al*, 2005; Limburg *et al*, 2006; Gunter *et al*, 2008; Stocks *et al*, 2011).

Our results from an older population are most similar to those of Schoen *et al* (1999), also from an older population, and those of Trevisan *et al* (2001), which showed a nearly two-fold increased risk of colorectal cancer for the highest *vs* lowest quartile of glucose (Schoen *et al*, 1999) and the highest quartile *vs* everyone else (Trevisan *et al*, 2001) in men and women combined. Our findings also appear to be consistent with those of Flood *et al* (2007), who found that fasting serum insulin and glucose were associated with increased risk of recurrent colorectal adenomas, an established preneoplastic lesion of the colon or rectum. In that study, the association of glucose, but not insulin, was most apparent for advanced adenomas. Furthermore, as in our analysis, previous studies have consistently shown stronger associations of markers of insulin resistance with colon cancer and adenoma compared with rectal cancer and adenoma (Giovannucci, 1995).

Differences in the results of studies to date may have a variety of explanations, including the unavailability of fasting bloods in some studies (Nilsen and Vatten, 2001; Palmqvist *et al*, 2003; Saydah *et al*, 2003; Tsushima *et al*, 2005), sample size (54 cases, Trevisan *et al*, 2001, to 4695 cases, Stocks *et al*, 2011), length of follow-up (3.4 years, Palmqvist *et al*, 2003 to 19 years Tsushima *et al*, 2005), differences in the age and sex distribution and the prevalence of other risk factors among studies, and adjustment for important covariates.

In a previous study conducted within the Women's Health Initiative Observational Study (Gunter *et al*, 2008), we showed that serum insulin, waist circumference, and free IGF-1 were each positively associated with colorectal cancer incidence in multivariate models. However, these associations became non-significant when adjusted for one another. Glucose was not associated with risk in that study. However, a number of differences between the earlier study and the present one may help account for the discrepancies in their results. First, the earlier study was a case-cohort study conducted in the WHI OS and had a larger sample size (438 colorectal cancer cases) and measured a number of hormones, including insulin, IGF-1, and estradiol. The present study was conducted in the subsample of women whose blood samples at multiple time points had been analysed for a number of ‘core analytes,’ including insulin and glucose. Women in the latter study were predominantly from the CT (81%). It should be noted that mean weight, BMI, and waist circumference at baseline were markedly greater in CT participants compared with OS participants (76.1 *vs* 71.7 kg; 28.9 *vs* 27.3 kg m^−2^; and 88.8 *vs* 84.8 cm—*P*<0.0001 for all comparisons). The fact that the association of glucose with colorectal cancer was somewhat strengthened when the analysis was restricted to women in the CT is consistent with the possibility that differences between the CT and OS populations may in part account for the differences in results between the two studies.

The attenuation of the initial association with insulin (Gunter *et al*, 2008) and, in another study, with glucose (Limburg *et al*, 2006), when other covariates were controlled for, indicates the difficulty of isolating a single primary factor from a cluster of highly correlated behavioural and metabolic variables, including body mass index, waist circumference, diabetes, physical activity, insulin, glucose, triglycerides, and IGF-1. The existence of a number of correlated factors suggests that it might be more appropriate to examine a cluster of risk factors. Trevisan *et al* (2001) reported that in a pooling of cohorts from Italy, subjects with relatively high levels of blood glucose and a cluster of metabolic abnormalities linked to insulin resistance experienced an increased risk. The HR for presence of the cluster of metabolic abnormalities (including hypertension, elevated glucose and triglycerides, and low HDL cholesterol) was 2.99, 95% CI 1.27–7.01, compared with 1.80 (1.05–3.09) for glucose alone. In general, studies which have examined the association of the metabolic syndrome, using a variety of definitions, with risk of colorectal cancer and adenoma have reported evidence of a positive association, and the consistency of the findings has been judged ‘remarkable’ (Giovannucci, 1995).

While insulin resistance is generally believed to be the underlying factor accounting for the associations of adiposity, physical inactivity, and metabolic variables with colorectal cancer, the biological mechanism involved is unknown (McKeown-Eyssen, 1994; Giovannucci, 1995; Strickler *et al*, 2001). Elevated levels of triglycerides, insulin, glucose, and IGF-1 have all been suggested as contributing to the association of the metabolic syndrome with increased risk of colorectal cancer (McKeown-Eyssen, 1994; Giovannucci, 1995). Although it is difficult to tease apart the contributions of these highly correlated factors, Giovannucci (1995) has argued that epidemiologic and animal experimental evidence point to a primary direct role of hyperinsulinaemia, rather than hyperglycaemia or other aspects of the insulin resistance syndrome in the development of colorectal cancer. However, we note that to date the epidemiologic evidence for a positive association of both fasting insulin and glucose with colorectal cancer risk in menopausal women is weak, but that for glucose may be somewhat stronger.

The present study benefited from the availability of repeated measurements of serum insulin and glucose over 12 years of follow-up. With one exception (Stocks *et al*, 2010), previous studies of the association of insulin and glucose with colorectal cancer risk have included only baseline measurements. Our time-dependent covariate analysis supported the existence of a statistically significant positive association with average glucose level during follow-up. Similar trends were observed when analysing measurements obtained during various time windows before the date of diagnosis of the cases, although the results were not statistically significant. Moreover, few studies have analysed HOMA-IR, a more direct measure of insulin resistance, which can only be computed using fasting insulin and glucose values, and which has been shown to reflect insulin resistance assessed by the euglycaemic clamp more accurately than fasting insulin alone (Ikeda *et al*, 2001). Other strengths of the present study include the central adjudication of diagnoses of colorectal cancer in WHI; the negligible loss to follow-up; and the availability of information on a wide range of potential confounding variables. A major limitation of our study is the relatively small number of cases, which limited our ability to examine associations with insulin and glucose by subsite within the colorectum, within strata of potential effect modifiers (e.g., age, body mass index and use of exogenous hormones), and in the time-dependent covariates analysis.

In conclusion, in this cohort study of postmenopausal women, elevated fasting serum glucose, but not insulin or HOMA-IR, was associated with roughly a two-fold increased risk of colorectal cancer. This association was robust and was not affected by adjustment for insulin or by exclusion of cases diagnosed in the first 2 years following enrollment. In time-dependent covariate analyses, trends using the average of all measurements showed a statistically significant positive association of glucose with colorectal and colon cancer risk. Our findings, together with those of several previous studies, suggest that insulin resistance may be related to increased risk of colorectal cancer, though the specific biologic mechanism remains to be elucidated.

## Figures and Tables

**Table 1:** Baseline characteristics of colorectal cancer cases and non-cases in the Women's Health Initiative

	**Cases (*n*=81)**	**Non-cases (*n*=4821)**	***P*-value**
Age (years)^a^	64.3±6.7	62.5±7.1	0.01
Body mass index (kg m^−2^)^a^	28.0±4.7	28.7±5.9	0.21
Waist circumference (cm)^a^	86.7±11.6	87.4±13.5	0.64
Height (cm)^a^	160.8±6.7	160.9±6.8	0.85
Parity^a^	2.9±1.7	2.7±1.7	0.41
Age at menopause^a^	47.5±6.6	47.0±6.8	0.39
Alcohol (servings per week)^a^	1.4±3.0	1.8±4.1	0.17
Physical activity (METs per week^b^)^a^	7.8±9.1	10.2±13.2	0.02
Oral contraceptive use (% ever)	39.5	42.2	0.63
Hormone therapy use (% ever)	50.6	47.9	0.63
Age at menarche (% ⩽12 years)	40.8	46.7	0.48
Age at first birth (% ⩾30 years)	8.8	9.0	0.68
			
*Colorectal cancer in a first degree*			
family member (% yes)	18.5	14.2	0.27
			
Education (% some post college)	23.5	25.0	0.92
Ethnicity (% non-Hispanic white)	65.4	54.0	0.09
Smoking (% current smokers)	9.9	8.1	0.54

a Mean (s.d.).

b METs, metabolic equivalent tasks (defined as caloric need per kilogram of body weight per hour of activity divided by the caloric need per kilogram of body weight per hour at rest) per hour per week.

**Table 2:** Association of baseline serum insulin, glucose, and HOMA-IR with risk of colorectal and colon cancer in the Women's Health Initiative

	**Colorectal cancer**							**Colon cancer**			
	***N* cases**	***N* non-cases**	**HR^a^**	**95% CI**	**HR^b^**	**95% CI**	***N* cases**	**HR^a^**	**95% CI**	**HR^b^**	**95% CI**
*Insulin* (μ*IU ml*^−*1*^*)*^c^											
<7.75	29	1561	1.00	Ref.	1.00	Ref.	21	1.00	Ref.	1.00	Ref.
7.75 to <11.85	24	1545	0.85	0.50–1.47	0.89	0.51–1.55	25	1.03	0.56–1.89	1.11	0.59–2.08
⩾11.85	27	1563	0.99	0.59–1.68	1.11	0.61–2.01	18	1.11	0.61–2.02	1.28	0.65–2.53
*P* trend			*0.99*		*0.75*			*0.73*		*0.48*	
Continuous (per 1 mg dl^−1^)					1.000	0.965–1.036				1.003	0.964–1.044
											
*Glucose (mg dl* ^*−1*^ *)*											
<89.5	18	1520	1.00	Ref.	1.00	Ref.	15	1.00	Ref.	1.00	Ref.
89.5 to <99.5	28	1641	1.41	0.78–2.57	1.32	0.72–2.40	28	1.84	0.92–3.68	1.72	0.85–3.48
⩾99.5	35	1660	1.73	0.98-3.06	1.74	0.97–3.15	22	2.18	1.11–4.29	2.25	1.12–4.51
*P* trend			*0.06*		*0.06*			*0.03*		*0.02*	
Continuous (per 1 mg dl^−1^)					1.031	1.009–1.054				1.034	1.009–1.060
											
*HOMA-IR*											
<31.2	27	1556	1.00	Ref.	1.00	Ref.	21	1.00	Ref.	1.00	Ref.
31.2 to <50.2	23	1560	0.86	0.50–1.51	0.93	0.52–1.66	24	1.02	0.55–1.89	1.14	0.60–2.16
⩾50.2	30	1553	1.18	0.70–1.98	1.37	0.76–2.49	19	1.27	0.70–2.29	1.51	0.77–2.98
*P* trend			0.54		*0.30*			*0.43*		*0.23*	
Continuous					1.001	0.995–1.007				1.002	0.995–1.009

Abbreviations: HR=hazard ratio; CI=confidence interval; HOMA-IR=homeostasis model assessment-insulin resistance; MET=metabolic equivalent task; OS=observational study.

a Adjusted for age.

b Adjusted for age, body mass index (kg m^−2^), alcohol intake (drinks per week), physical activity (MET-hours per week), family history of colorectal cancer, ethnicity (white, black, other), and participation in the OS or treatment arm of each clinical trial.

c One case and 152 non-cases were missing a baseline insulin value.

## References

[bib1] Bergmeyer HU (ed.) (1974) Methods of Enzymatic Analysis. 2nd Engl ed., p 1196. Academic Press: New York

[bib2] Bjork J, Nilsson J, Hultcrantz R, Johansson C (1993) Growth-regulatory effects of sensory neuropeptides, epidermal growth factor, insulin, and somatostatin on the non-transformed intestinal epithelial cell line IEC-6 and the colon cancer cell line HT 29. Scand J Gastroenterol 28(10): 879–884 7505479 10.3109/00365529309103129

[bib3] Bruce WR, Corpet DE (1996) The colonic protein fermentation and insulin resistance hypotheses for colon cancer etiology: experimental tests using precursor lesions. Eur J Cancer Prev 5(Suppl 2): 41–47 10.1097/00008469-199612002-00007 9061294

[bib4] Chlebowski RT, Chen Z, Anderson GL, Rohan T, Aragaki A, Lane D, Dolan NC, Paskett ED, McTiernan A, Hubbell FA, Adams-Campbell LL, Prentice R (2005) Ethnicity and breast cancer: factors influencing differences in incidence and outcome. J Natl Cancer Inst 97(6): 439–448 15770008 10.1093/jnci/dji064

[bib5] Curb JD, McTiernan A, Heckbert SR, Kooperberg C, Stanford J, Nevitt M, Johnson KC, Proulx-Burns L, Pastore L, Criqui M, Daugherty S (2003) Outcomes ascertainment and adjudication methods in the Women's Health Initiative. Ann Epidemiol 13(5): S122–S128 14575944 10.1016/s1047-2797(03)00048-6

[bib6] Flood A, Mai V, Pfeiffer R, Kahle L, Remaley AT, Lanza E, Schatzkin A (2007) Elevated serum concentrations of insulin and glucose increase risk of recurrent colorectal adenomas. Gastroenterology 133(5): 1423–1429 17904132 10.1053/j.gastro.2007.08.040

[bib7] Gail MH (1981) Evaluating serial cancer marker studies in patients at risk of recurrent disease. Biometrics 37(1): 67–78 7248444

[bib8] Giovannucci E (1995) Insulin and colon cancer. Cancer Causes Control 6(2): 164–179 7749056 10.1007/BF00052777

[bib9] Gunter MJ, Hoover DR, Yu H, Wassertheil-Smoller S, Rohan TE, Manson JE, Howard BV, Wylie-Rosett J, Anderson GL, Ho GY, Kaplan RC, Li J, Xue X, Harris TG, Burk RD, Strickler HD (2008) Insulin, insulin-like growth factor-I, endogenous estradiol, and risk of colorectal cancer in postmenopausal women. Cancer Res 68(1): 329–337 18172327 10.1158/0008-5472.CAN-07-2946 PMC4225702

[bib10] Howard BV, Adams-Campbell L, Allen C, Black H, Passaro M, Rodabough RJ, Rodriguez BL, Safford M, Stevens VJ, Wagenknecht LE (2004) Insulin resistance and weight gain in postmenopausal women of diverse ethnic groups. Int J Obes Relat Metab Disord 28(8): 1039–1047 15254486 10.1038/sj.ijo.0802645

[bib11] Huxley RR, Ansary-Moghaddam A, Clifton P, Czernichow S, Parr CL, Woodward M (2009) The impact of dietary and lifestyle risk factors on risk of colorectal cancer: a quantitative overview of the epidemiological evidence. Int J Cancer 125(1): 171–180 19350627 10.1002/ijc.24343

[bib12] Ikeda Y, Suehiro T, Nakamura T, Kumon Y, Hashimoto K (2001) Clinical significance of the insulin resistance index as assessed by homeostasis model assessment. Endocr J 48(1): 81–86 11403106 10.1507/endocrj.48.81

[bib13] Jee SH, Ohrr H, Sull JW, Yun JE, Ji M, Samet JM (2005) Fasting serum glucose level and cancer risk in Korean men and women. JAMA 293(2): 194–202 15644546 10.1001/jama.293.2.194

[bib14] Jenab M, Riboli E, Cleveland RJ, Norat T, Rinaldi S, Nieters A, Biessy C, Tjonneland A, Olsen A, Overvad K, Gronbaek H, Clavel-Chapelon F, Boutron-Ruault MC, Linseisen J, Boeing H, Pischon T, Trichopoulos D, Oikonomou E, Trichopoulou A, Panico S, Vineis P, Berrino F, Tumino R, Masala G, Peters PH, van Gils CH, Bueno-de-Mesquita HB, Ocke MC, Lund E, Mendez MA, Tormo MJ, Barricarte A, Martinez-Garcia C, Dorronsoro M, Quiros JR, Hallmans G, Palmqvist R, Berglund G, Manjer J, Key T, Allen NE, Bingham S, Khaw KT, Cust A, Kaaks R (2007) Serum C-peptide, IGFBP-1 and IGFBP-2 and risk of colon and rectal cancers in the European Prospective Investigation into Cancer and Nutrition. Int J Cancer 121(2): 368–376 17372899 10.1002/ijc.22697

[bib15] Kaaks R, Toniolo P, Akhmedkhanov A, Lukanova A, Biessy C, Dechaud H, Rinaldi S, Zeleniuch-Jacquotte A, Shore RE, Riboli E (2000) Serum C-peptide, insulin-like growth factor (IGF)-I, IGF-binding proteins, and colorectal cancer risk in women. J Natl Cancer Inst 92(19): 1592–1600 11018095 10.1093/jnci/92.19.1592

[bib16] Koenuma M, Yamori T, Tsuruo T (1989) Insulin and insulin-like growth factor 1 stimulate proliferation of metastatic variants of colon carcinoma 26. Jpn J Cancer Res 80(1): 51–58 2540132 10.1111/j.1349-7006.1989.tb02244.x PMC5917674

[bib17] Limburg PJ, Stolzenberg-Solomon RZ, Vierkant RA, Roberts K, Sellers TA, Taylor PR, Virtamo J, Cerhan JR, Albanes D (2006) Insulin, glucose, insulin resistance, and incident colorectal cancer in male smokers. Clin Gastroenterol Hepatol 4(12): 1514–1521 17162243 10.1016/j.cgh.2006.09.014 PMC1766481

[bib18] Ma J, Giovannucci E, Pollak M, Leavitt A, Tao Y, Gaziano JM, Stampfer MJ (2004) A prospective study of plasma C-peptide and colorectal cancer risk in men. J Natl Cancer Inst 96(7): 546–553 15069117 10.1093/jnci/djh082

[bib19] Matthews DR, Hosker JP, Rudenski AS, Naylor BA, Treacher DF, Turner RC (1985) Homeostasis model assessment: insulin resistance and beta-cell function from fasting plasma glucose and insulin concentrations in man. Diabetologia 28(7): 412–419 3899825 10.1007/BF00280883

[bib20] McKeown-Eyssen G (1994) Epidemiology of colorectal cancer revisited: are serum triglycerides and/or plasma glucose associated with risk? Cancer Epidemiol Biomarkers Prev 3(8): 687–695 7881343

[bib21] Moghaddam AA, Woodward M, Huxley R (2007) Obesity and risk of colorectal cancer: a meta-analysis of 31 studies with 70 000 events. Cancer Epidemiol Biomarkers Prev 16(12): 2533–2547 18086756 10.1158/1055-9965.EPI-07-0708

[bib22] Nilsen TI, Vatten LJ (2001) Prospective study of colorectal cancer risk and physical activity, diabetes, blood glucose and BMI: exploring the hyperinsulinaemia hypothesis. Br J Cancer 84(3): 417–422 11161410 10.1054/bjoc.2000.1582 PMC2363734

[bib23] Ning Y, Wang L, Giovannucci EL (2010) A quantitative analysis of body mass index and colorectal cancer: findings from 56 observational studies. Obes Rev 11(1): 19–30 19538439 10.1111/j.1467-789X.2009.00613.x

[bib24] Otani T, Iwasaki M, Sasazuki S, Inoue M, Tsugane S (2007) Plasma C-peptide, insulin-like growth factor-I, insulin-like growth factor binding proteins and risk of colorectal cancer in a nested case-control study: the Japan public health center-based prospective study. Int J Cancer 120(9): 2007–2012 17266031 10.1002/ijc.22556

[bib25] Palmqvist R, Stattin P, Rinaldi S, Biessy C, Stenling R, Riboli E, Hallmans G, Kaaks R (2003) Plasma insulin, IGF-binding proteins-1 and -2 and risk of colorectal cancer: a prospective study in northern Sweden. Int J Cancer 107(1): 89–93 12925961 10.1002/ijc.11362

[bib26] Peterson JI, Young DS (1968) Evaluation of the hexokinase-glucose-6-phosphate dehydrogenase method of determination of glucose in urine. Anal Biochem 23(2): 301–316 5657801 10.1016/0003-2697(68)90361-8

[bib27] Saydah SH, Platz EA, Rifai N, Pollak MN, Brancati FL, Helzlsouer KJ (2003) Association of markers of insulin and glucose control with subsequent colorectal cancer risk. Cancer Epidemiol Biomarkers Prev 12(5): 412–418 12750235

[bib28] Schoen RE, Tangen CM, Kuller LH, Burke GL, Cushman M, Tracy RP, Dobs A, Savage PJ (1999) Increased blood glucose and insulin, body size, and incident colorectal cancer. J Natl Cancer Inst 91(13): 1147–1154 10393723 10.1093/jnci/91.13.1147

[bib29] Stocks T, Lukanova A, Bjorge T, Ulmer H, Manjer J, Almquist M, Concin H, Engeland A, Hallmans G, Nagel G, Tretli S, Veierod MB, Jonsson H, Stattin P (2011) Metabolic factors and the risk of colorectal cancer in 580 000 men and women in the metabolic syndrome and cancer project (Me-Can). Cancer 117: 2398–2407 24048787 10.1002/cncr.25772

[bib30] Strickler HD, Wylie-Rosett J, Rohan T, Hoover DR, Smoller S, Burk RD, Yu H (2001) The relation of type 2 diabetes and cancer. Diabetes Technol Ther 3(2): 263–274 11478333 10.1089/152091501300209633

[bib31] The Women's Health Initiative Study Group (1998) Design of the Women's Health Initiative clinical trial and observational study. Control Clin Trials 19(1): 61–109 9492970 10.1016/s0197-2456(97)00078-0

[bib32] Tietz NW. (ed) (1987) Fundamentals of Clinical Chemistry 3rd ed., p 544. WB Saunders Co: Philadelphia, PA

[bib33] Trevisan M, Liu J, Muti P, Misciagna G, Menotti A, Fucci F (2001) Markers of insulin resistance and colorectal cancer mortality. Cancer Epidemiol Biomarkers Prev 10(9): 937–941 11535544

[bib34] Tsushima M, Nomura AM, Lee J, Stemmermann GN (2005) Prospective study of the association of serum triglyceride and glucose with colorectal cancer. Dig Dis Sci 50(3): 499–505 15810632 10.1007/s10620-005-2464-5

[bib35] Warburg O (1956) On the origin of cancer cells. Science 123(3191): 309–314 13298683 10.1126/science.123.3191.309

[bib36] Wei EK, Ma J, Pollak MN, Rifai N, Fuchs CS, Hankinson SE, Giovannucci E (2005) A prospective study of C-peptide, insulin-like growth factor-I, insulin-like growth factor binding protein-1, and the risk of colorectal cancer in women. Cancer Epidemiol Biomarkers Prev 14(4): 850–855 15824155 10.1158/1055-9965.EPI-04-0661

[bib37] Werner H, LeRoith D (1996) The role of the insulin-like growth factor system in human cancer. Adv Cancer Res 68: 183–223 8712068 10.1016/s0065-230x(08)60354-1

[bib38] Wu X, Fan Z, Masui H, Rosen N, Mendelsohn J (1995) Apoptosis induced by an anti-epidermal growth factor receptor monoclonal antibody in a human colorectal carcinoma cell line and its delay by insulin. J Clin Invest 95(4): 1897–1905 7706497 10.1172/JCI117871 PMC295734

[bib39] Yamada K, Araki S, Tamura M, Sakai I, Takahashi Y, Kashihara H, Kono S (1998) Relation of serum total cholesterol, serum triglycerides and fasting plasma glucose to colorectal carcinoma *in situ*. Int J Epidemiol 27(5): 794–798 9839735 10.1093/ije/27.5.794

